# Post-activation performance enhancement of flywheel and traditional squats on vertical jump under individualized recovery time

**DOI:** 10.3389/fphys.2024.1443899

**Published:** 2024-09-06

**Authors:** Shaocheng Sun, Ying Yu, Yu Niu, Meiling Ren, Jiaoqin Wang, Ming Zhang

**Affiliations:** ^1^ China Volleyball College, Beijing Sport University, Beijing, China; ^2^ Key Laboratory of Sport Training of General Administration of Sport of China, Beijing Sport University, Beijing, China; ^3^ Institute of Physical Education and Training, Capital University of Physical Education and Sports, Beijing, China

**Keywords:** flywheel training, eccentric overload, warm-up, load-velocity profile, velocity-based training, sports fatigue, jumping potentiation

## Abstract

**Purpose:**

To explore the post-activation performance enhancement (PAPE) of flywheel and traditional squats on a series of vertical jumps, the loads of the two protocols were matched based on their linear velocities. In addition, we attempted to validate the effectiveness of determining individualized recovery time (IRT) between conditioning activities and explosive movements.

**Methods:**

Sixteen trained players participated in three main experiments: first, one-repetition maximum (1RM) assessment and intensity matching test; second, the weighted jump squat (WJS) test at baseline and at 2, 4, 6, 8, and 10 min after flywheel and traditional protocols; and third, squat jump (SJ), countermovement jump (CMJ), and approach jump (AJ) tests incorporating IRT determined in the WJS sessions into both protocols. These protocols were standardized to 8 repetitions at 80% 1RM with equivalent concentric speed matched by a linear position transducer and conducted in a random order on separate days.

**Results:**

In the WJS tests, both protocols exhibited significant increases on jump height (JH), peak force (PF), and peak power (PP) after 2 to 6 min (all *p* < 0.05), and the time courses of changes in performance were in a similar trend. In the SJ, CMJ, and AJ tests, both protocols demonstrated highly significant increases on JH, PP, and reactive strength index (RSI) after incorporating IRT (all *p* < 0.01), with all participants exhibiting diverse improvement above the baseline levels. The potentiation percentages of the flywheel protocol on JH, PP, and RSI were higher than those of the traditional protocol across four jumping types (JH: 5.35%–9.79% vs. 4.13%–8.46%; PP: 4.16%–6.13% vs. 3.23%–4.77%; and RSI: 7.27% vs. 7.04%).

**Conclusion:**

High-intensity flywheel squats can produce jumping potentiation in neuromechanical factors comparable to, or even surpassing, those observed in traditional squats, potentially making them a more effective option for inducing PAPE. Additionally, incorporating IRT into potentiation protocols could further optimize the PAPE effects.

## 1 Introduction

Post-activation performance enhancement (PAPE) is the phenomenon in which neuromuscular voluntary activities (VAs) are acutely enhanced by contractile history (conditioning activities, CAs). The mechanism of PAPE is related to physiological phosphorylation of myosin regulatory light chains, as well as increases in muscle temperature, muscle blood flow, neural drive, and muscle–tendon stiffness (Blazevich and Babault, 2019; Tillin and Bishop, 2009). PAPE becomes the premise of complex training and warm-up routines due to its significant effects on physical performance (Cuenca-Fernández et al., 2017; Robbins, 2005). Consensus is gradually emerging in some aspects of PAPE research, whereas others remain equivocal. The recommendations for the optimal rest interval between CAs and VAs showed variation from 3 to 12 min (Dobbs et al., 2019; Gouvêa et al., 2013). This discrepancy is likely attributed to participants’ intra-complex responses and the clustering of multiple effects within a single study. Higher strength level individual appears to express PAPE earlier than weaker individual (Seitz et al., 2014). If CA has sufficient intensity and relatively low volume, potentiation also may be realized earlier (Tillin and Bishop, 2009). However, Naclerio et al. (2015) examined the effects of three different volumes of CAs and revealed no significant correlation between the volume of CAs and the optimal recovery time. Such studies highlight the multifaceted and complex nature of PAPE, underscoring the need of individualized recovery time (IRT) to fully realized potential benefits of PAPE.

Vertical jump ability is crucial for success in many sports, and potentiation protocols for lower body have been frequently investigated (Dobbs et al., 2019; Suchomel et al., 2016). These protocols are designed to enhance subsequent performance in various vertical jumps, executed from a static start, countermovement action, approach, *etc*. However, CA generates a dual response in muscles, inducing both fatigue and PAPE (Sale, 2002). The dynamic balance between these two responses changes over recovery time, which is influenced by a series of external factors such as the intensity, volume, and motor pattern of CAs, as well as internal factors including the participant’s strength level, fiber-type distribution, and power–strength ratio (Seitz and Haff, 2016; Wilson et al., 2013). Therefore, identifying and optimizing these variables is essential for jumping potentiation.

Squat is one of the most popular and important exercises for the development of strength and vertical jump ability, and is considered an essential tool in strength and conditioning training programming. Considering the portability of devices, traditional squat might not be the preferred modality to induce PAPE. Flywheel training (FT), which provides inertial resistance and eccentric overload through spinning flywheels, has recently become increasingly popular due to its ease of use in a variety of sporting applications (Berg and Tesch, 1998; Petré et al., 2018). FT could produce greater force and muscle activation in eccentric than concentric activities through the kinetic energy stored in the flywheel (Norrbrand et al., 2010), and increase elastic energy storage in the muscle–tendon unit to amplify the potential of stretch-shortening cycle (SSC) (Komi, 1986). Nardone et al. (1989) observed the selective recruitment of high-threshold motor units during eccentric contractions. Similarly, Fang et al. (2001) found greater amplitude for the movement-related cortical potential during the eccentric phase relative to the concentric phase. It is reported that FT has been widely applied to inducing acute and chronic adaptions in numerous sports (Cuenca-Fernández et al., 2019; Tous-Fajardo et al., 2016). However, few studies compared FT to traditional exercise. This could be attributed to the difficulty in matching intensities between them, as FT uses inertial load instead of gravitational load. Nonetheless, velocity-based training (VBT) provides a means to match intensity based on speed, such as providing feedback on mean velocity during concentric and eccentric action, possibly bridging the gap in comparative research (Carroll et al., 2019; Weakley et al., 2021).

Given this context, the main purposes of this study were twofold. First, it aimed to investigate the PAPE effects of flywheel and traditional squats on vertical jump performance based on the same CA intensity and volume, with weighted jump squat (WJS) serving as the VA type. Second, it aimed to examine the effectiveness of IRT in optimizing PAPE by using the same CA and setting SJ, CMJ, and AJ as VA types. We hypothesized that flywheel squats would exhibit greater effects of jumping potentiation than traditional squats, and applying IRT to protocols would further improve the PAPE effects.

## 2 Methods

### 2.1 Participants

Sixteen elite male volleyball players from Beijing Sport University with at least 4 years of resistance training history (age: 22.3 [1.3] y; height: 188.4 [6.2] cm; body mass: 81.2 [8.1] kg) gave their written informed consent to participate in this study. Values are expressed as mean [SD]. All participants had at least 3 years of competition experience at the national level and were prominent members of the university’s elite team. The sample size was calculated using G*Power software (version 3.1). We selected the “F tests–ANOVA: repeated measures, within factors” for the statistical analysis. The *a priori* estimation of the minimum sample size was based on alpha, power, and effect size. Using an effect size of *f* = 0.3, an alpha level of 0.05, and a power of 0.8, with the number of measurements set to 6, the analysis indicated a required sample size of 14 participants. To account for potential attrition, we recruited two participants in addition. The research received approval from the Beijing Sport University Human Participants Review Board (no. 2023193H), ensuring adherence to the principles of the Declaration of Helsinki.

### 2.2 Design and procedures

**FIGURE 1:**
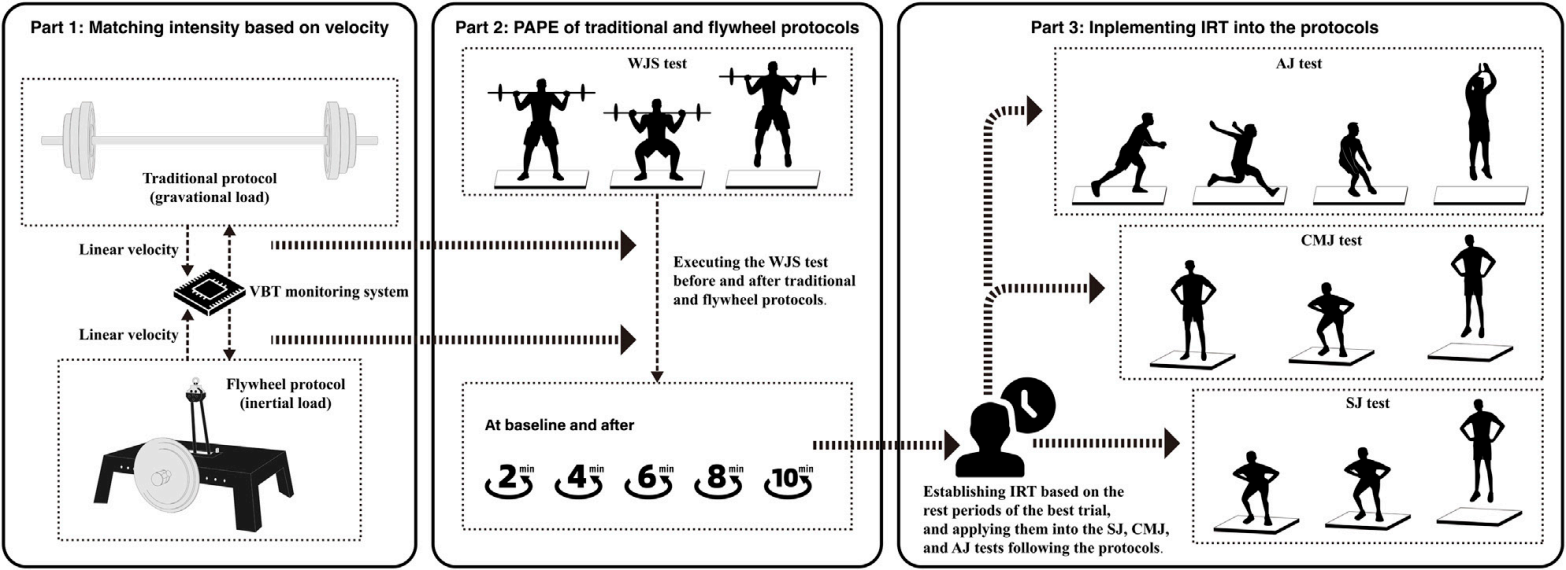
Experimental procedure diagram.

A randomized and counterbalanced crossover design was used to compare the PAPE effects of flywheel squats and traditional squats on vertical jumps. Participants attended the laboratory on 13 separate days over a 5- to 6-week period, including 3 familiarization visits to achieve an acceptable level of technical proficiency, 1RM assessment and intensity matching test as preliminary experiments, 2 WJS sessions with the flywheel and traditional protocols, and 2 sessions for each SJ, CMJ, and AJ test with IRT implemented into both protocols (Figure 1). To minimize the sources of bias, participants were scheduled to attend the laboratory at the same time (±2 h) in each day of experiments, and they were instructed not to engage in any strenuous lower limb exercises in the preceding 48 h.

#### 2.2.1 1RM assessment and intensity matching test

Once participants arrived at the laboratory, they executed a standardized warm-up procedure, including 6 min of pedaling on a cycle ergometer (Monark 828E cycle ergometer; Vansbro, Dalarna, Sweden) at 80 W and 60 revolutions per minute, 3 min of dynamic stretching, and 10 full-depth bodyweight squats. During the first session, participants commenced the 1RM assessment, comprising sets estimated at 60% 1RM (6 repetitions) and 80% 1RM (3 repetitions). Subsequently, five attempts were permitted to establish individual’s 1RM, and the barbell load was increased progressively from 5% to 10% until the 1RM was achieved (Haff and Triplett, 2015). Two minutes passive rest was allocated between all warm-up sets and 3 minutes between 1RM attempts. After the 1RM assessment, participants were given 5 min of rest. Then, they performed barbell squats with the maximal concentric effort using 80% 1RM (8 repetitions), and a linear position transducer (Tendo Unit, London, United Kingdom) was attached to the barbell, measuring the mean concentric linear velocity (MCLV)—average velocity during the propulsive phase of each lift. In the second session, after the standardized warm-up, participants performed flywheel squat (10 repetitions) on a flywheel device (Desmotec D.FULL, Biella, Italy), using four inertial loads in a randomized order (0.1176 kg·m^2^, 0.1568 kg·m^2^, 0.1960 kg·m^2^, and 0.2352 kg·m^2^), with 5 min passive recovery given between sets. A PVC pipe was placed on each participant’s shoulders, in a manner similar to that of barbell squats, and velocity data were collected using linear position transducer. In each set, the first two repetitions served solely to initiate the movement, and the MCLV was calculated from eight repetitions with the maximal concentric effort.

#### 2.2.2 WJS sessions with IRT establishing

**FIGURE 2:**
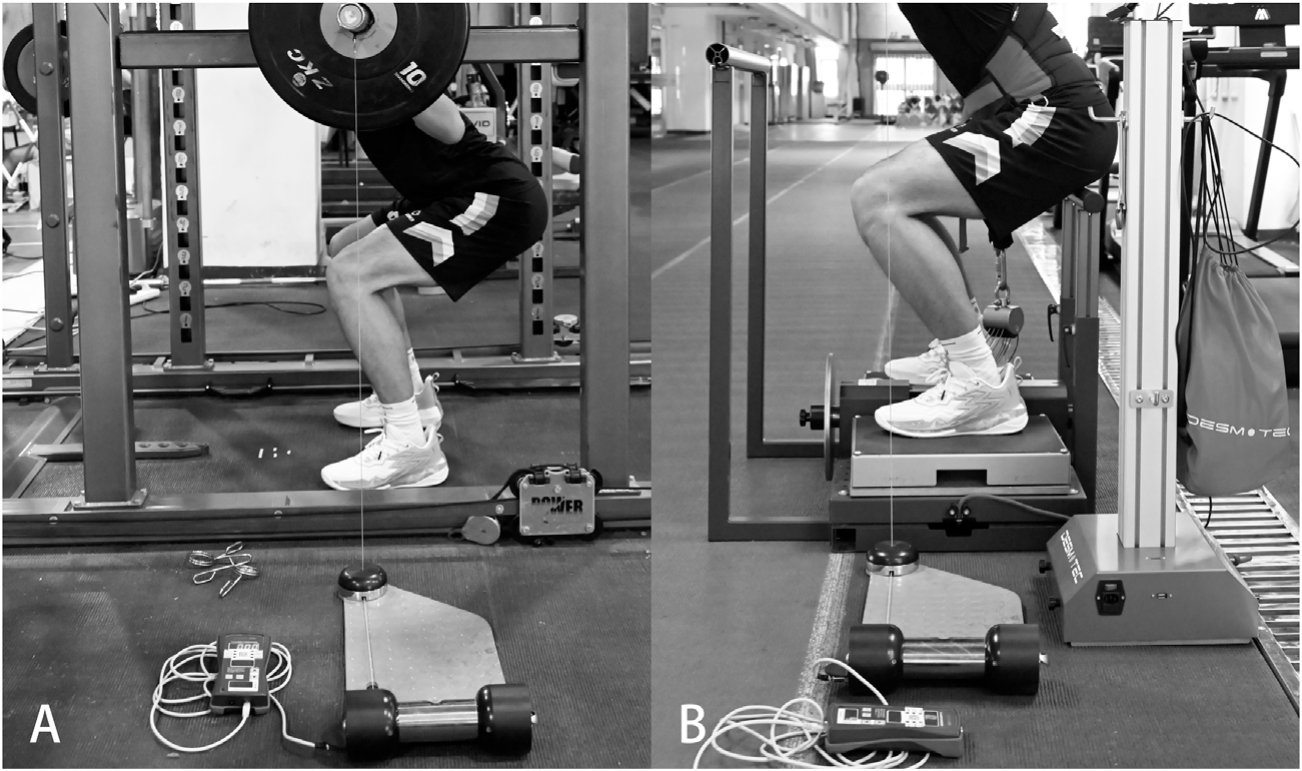
Experimental setups for the traditional protocol **(A)** and the flywheel protocol **(B)**.

The intervals at which individuals reached their peak flight height were established as their IRT. Flywheel and traditional protocols were randomly arranged across two WJS sessions. Prior to the experimental trials, participants executed the standardized warm-up as previously described. Following a 5-min rest, participants executed the baseline test, consisting of two repetitions of WJS loaded with barbell weight at 15% 1RM and separated by 30 s. This approach was designed to provide sufficient load to stimulate the power output while ensuring that subsequent tests remain unaffected (Kirby et al., 2010; Zhang et al., 2023). During each repetition, participants squatted down to a self-selected depth and then immediately jumped as high as possible. The baseline value was determined by the average of repetitions. Kinetic data collection and analysis were conducted using a force plate (Kistler 9287, Winterthur, Switzerland) at a sampling frequency of 1000 Hz using BioWare software (Type 2182). After the baseline test, a 5-min rest was given, and then the participants performed either a barbell squat at 80% 1RM (8 repetitions) or flywheel squat (8 repetitions, excluding initial 2 repetitions) with the equivalent MCLV determined by individual’s load–velocity profile. The pins and seat were set to maintain the same squat depth at knee flexion of 100° across different protocols (Schoenfeld, 2010). Figures 2A,B demonstrate the experimental settings for both protocols. Visual feedback of MCLV and verbal encouragement were provided during each repetition to maintain the prescribed intensity. Following potentiation protocols, the post-test was performed in the same manner as the pre-test, repeated at intervals of 2, 4, 6, 8, and 10 min. The intervals at which individuals reached their peak flight height were established as their IRT.

#### 2.2.3 SJ, CMJ, and AJ sessions with IRT validating

Each session for SJ, CMJ, and AJ were similar to WJS session, maintaining consistent procedural structure in standardized warm-up, pre-test, both potentiation protocols, and post-test. However, there were altered variables in terms of jumping type and rest interval. Instead of fixed rest intervals, the IRT for each participant was utilized between potentiation protocols and jumping tests. In the SJ test, participants began in a position at 90° knee flexion and hands on the pelvic spine. From this static pose, they executed a rapid upward vertical jump as high as possible. In the CMJ test, participants started standing upright with hands on the pelvic spine and then quickly moved downward followed by an immediate upward vertical jump in a continuous motion. In the AJ test, participants took 2 to 3 steps forward, followed by a rapid upward vertical jump accompanied by a forceful arm swing. Participants were advised to execute AJ in a manner most proficient to them because of imposing further standardization in the jumping procedure could potentially hinder the fully experienced athletes’ performance.

#### 2.2.4 Kinematic and kinetic parameters

The analysis of force–time data in jumping tests involved several neuromechanical parameters, such as jump height (JH), peak force (PF), peak power (PP), average rate of force development (ARFD), and reactive strength index (RSI). The JH for WJS, SJ, and CMJ was determined using the impulse–momentum method, identifying the movement onset as the threshold of 5 SD below bodyweight. The JH for AJ was determined using the flight time method, and the threshold was defined as 5 SD of flight force (when the force plate is unloaded) (McMahon et al., 2018). The PF was defined as the maximum force observed on the force–time curve during jumping. The PP was determined by multiplying force with velocity, where velocity was deduced by integrating the force–time trace. The ARFD was calculated as the mean tangential slope from the initial point to the peak force (RFD = ΔForce/ΔTime). The point for SJ and CMJ was set at 5 SD above bodyweight, and AJ was forced to reach 5 SD unloaded value. The RSI was calculated as the ratio of flight height to contact time. Typical errors expressed as intraclass correlation coefficients, standard errors of measurement, and coefficients of variation (CV) are reported for kinematic and kinetic variables (Table 1).

**TABLE 1:** Typical errors of jumping tests for kinematic and kinetic variables.

Jumping test	Variable	ICC	SEM%	CV%
Weighted jump squat	JH (cm)	0.82	2.30	42.00
	PF (N)	0.93	1.34	46.28
	PP (W)	0.84	1.71	43.49
	ARFD (N·s^−1^)	0.91	8.67	73.26
Squat jump	JH (cm)	0.86	2.48	15.92
	PF (N)	0.82	1.64	47.73
	PP (W)	0.93	2.02	22.75
	ARFD (N·s^−1^)	0.81	7.33	50.80
Countermovement jump	JH (cm)	0.96	1.68	21.53
	PF (N)	0.95	1.58	44.07
	PP (W)	0.98	2.13	21.30
	ARFD (N·s^−1^)	0.83	7.98	47.28
Approach jump	JH (cm)	0.91	1.53	18.43
	PF (N)	0.87	1.71	46.97
	ARFD (N·s^−1^)	0.83	6.45	41.88
	RSI (m·s^−1^)	0.93	2.61	22.20

Abbreviations: ICC, intraclass correlation coefficient; SEM, standard error of measurement; CV, coefficient of variation; JH, jump height; PF, peak force; PP, peak power; ARFD, average rate of force development; RSI, reactive strength index.

#### 2.2.5 Statistical analysis

All statistical analyses were performed using SPSS v.27.0 (SPSS IBM, Chicago, IL, United States). The normality of distribution was tested using the Shapiro–Wilk test. A one-way within-subject analysis of variance (ANOVA) was employed for MCLV against the inertial load. Data variance was tested using Mauchly’s test of sphericity, and a Greenhouse–Geisser correction was applied when the assumption of sphericity was violated. A linear regression was performed using MCLV to predict the inertial load. A two-way repeated measures ANOVA was employed to compare the PAPE effects of protocols (traditional and flywheel squats) and time (baseline, 2, 4, 6, 8, and 10 min) on the performance of WJS. In cases where significant effects were observed in the ANOVAs, differences between conditions were identified using the Bonferroni *post hoc* pairwise comparisons. A paired-sample *t*-test was employed to compare differences in performance for SJ, CMJ, and AJ before and after implementing IRT into traditional and flywheel protocols. Cohen’s *d* effect sizes were assessed to determine meaningful differences, which are classified as trivial: <0.2, small: ≥0.2, medium: ≥0.5, and large: ≥0.8 (Cohen, 2013). The significance level was set at *p* < 0.05, and *p* < 0.01 was considered highly significant.

## 3 Results

**FIGURE 3:**
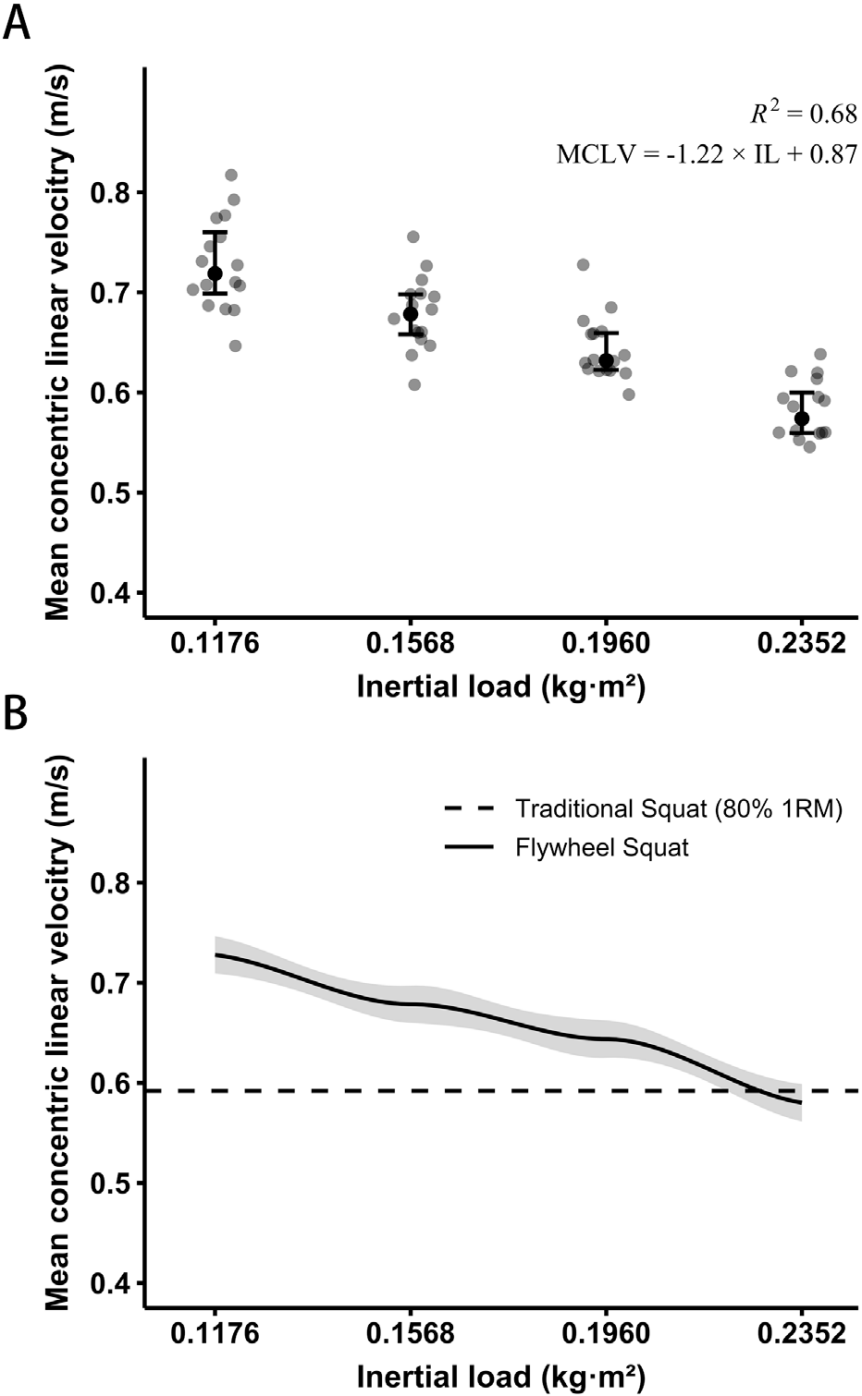
Mean concentric linear velocity (MCLV) of the flywheel squat at different inertial loads (IL) **(A)**. Error bars represent the upper and lower quartiles. An individual example of matching intensity between the two protocols based on the MCLV of 80% 1RM traditional squat **(B)**.

The one-way ANOVA was highly significant for MCLV (*p* < 0.001). Post hoc analyses revealed significant differences in MCLV across all inertial loads (*p* ≤ 0.047; 1.02 ≤ *d* ≤ 3.71). Additionally, a statistically significant regression equation MCLV = −1.22 × IL + 0.87 was observed (*F* (_3,45_) = 42.321; *p* < 0.001) with *R* ^2^ = 0.68 (Figure 3A). Based on individualizing linear regression models, each participant’s intensity was determined corresponding to the MCLV of the 80% 1RM traditional squat (Figure 3B).

**FIGURE 4:**
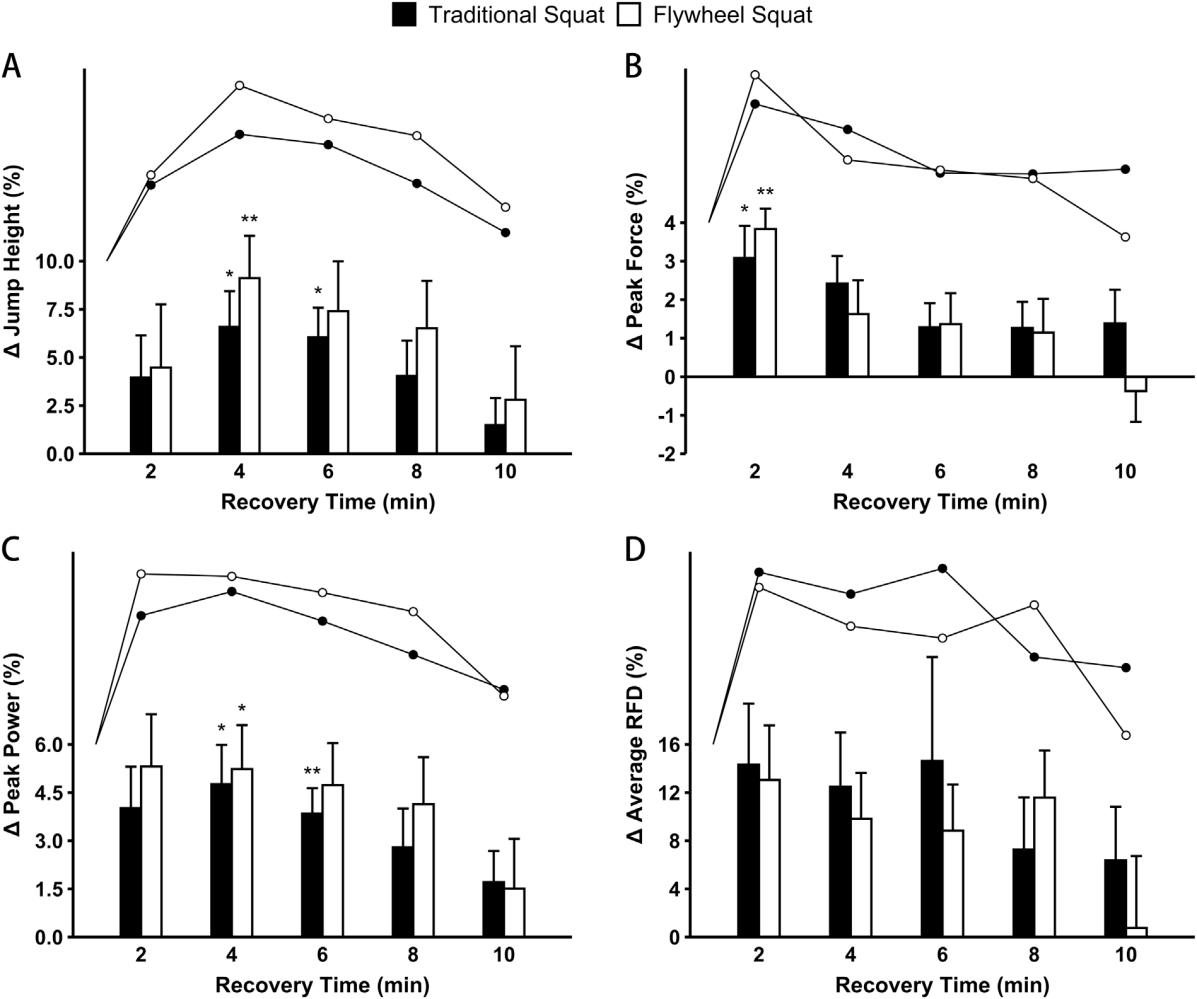
Percentage differences in the performance of weighted jump squats for jump height **(A)**, peak force **(B)**, peak power **(C)**, and average rate of force development (RFD) **(D)** during continuous time course after both protocols. The error bars represent SE. *Significant difference (*p* < 0.05) and **highly significant difference (*p* < 0.01) compared with baseline within each protocol.

In the WJS tests, there were significant main effects of time on JH, PF, PP, and ARFD (3.127 ≤ *F* (_5,75_) ≤ 10.804; *p* ≤ 0.021; 0.l8 ≤ *η*_*p*_^2^ ≤ 0.42). However, no significant main effects of protocols (*F* (_1,15_) ≤ 2.903; *p* ≥ 0.110; 0.01 ≤ *η*_*p*_^2^ ≤ 0.16) or interactional effects of protocol × time (*F* (_5,75_) ≤ 1.405; *p* ≥ 0.243; 0.01 ≤ *η*_*p*_^2^ ≤ 0.09) were found. Compared with the baselines, PAPE effects of both protocols were found on WJS (Table 2). After the flywheel protocol, significant improvements were observed in JH at 4 min (+9.13% [8.76%]; *p* = 0.003; *d* = 1.23) (Figure 4A), PF at 2 min (+3.84% [2.11%]; *p* < 0.001; *d* = 1.68) (Figure 4B), and PP at 4 min (+5.24% [5.46]; *p* = 0.027; *d* = 0.95) (Figure 4C). Following the traditional protocol, significant increases were observed in JH at 4 min (+6.59% [7.44%]; *p* = 0.034; *d* = 0.92) and 6 min (+6.05% [6.13%]; *p* = 0.020; *d* = 0.98) (Figure 4A), PF at 2 min (+3.08% [3.33%]; *p* = 0.040; *d* = 0.90) (Figure 4B), and PP at 4 min (+4.77% [4.89]; *p* = 0.032; *d* = 0.72) and 6 min (+3.84% [3.19]; *p* = 0.004; *d* = 0.92) (Figure 4C). Despite observing an upward trend in ARFD after following both protocols, the changes compared to the baselines were not statistically significant (*p* ≥ 0.278; −0.13 ≤ *d* ≤ 0.64) (Figure 4D).

**TABLE 2:** Effects of post-activation performance enhancement on weighted jump squats after flywheel and traditional protocols (mean ± SD).

Variable	Baseline	2 min	4 min	6 min	8 min	10 min
Flywheel protocol						
JH (cm)	27.42 ± 3.99	28.34 ± 3.33	29.67 ± 2.99 **	29.23 ± 3.62	28.99 ± 3.60	28.03 ± 4.19
PF (N)	2,167.08 ± 144.66	2,250.85 ± 168.41 **	2,203.67 ± 185.10	2,196.18 ± 156.82	2,191.74 ± 163.05	2,159.81 ± 170.48
PP (W)	4,677.04 ± 406.64	4,924.01 ± 520.07	4,920.06 ± 481.86 *	4,890.24 ± 396.69	4,864.04 ± 433.28	4,739.91 ± 426.02
ARFD (N·s^−1^)	2,724.76 ± 1,208.33	3,089.21 ± 1,668.71	2,973.60 ± 1,380.17	2,927.35 ± 1,268.00	3,004.82 ± 1,364.15	2,596.98 ± 1,100.64
Traditional protocol						
JH (cm)	27.87 ± 3.28	28.80 ± 2.71	29.59 ± 2.96 *	29.49 ± 3.27 *	29.01 ± 4.20	28.28 ± 3.61
PF (N)	2,169.73 ± 186.56	2,234.67 ± 181.15 *	2,219.97 ± 168.90	2,195.64 ± 171.58	2,195.82 ± 179.55	2,197.90 ± 180.08
PP (W)	4,678.52 ± 506.95	4,860.23 ± 531.44	4,895.71 ± 516.25 *	4,856.13 ± 525.05 **	4,812.05 ± 589.99	4,752.86 ± 492.21
ARFD (N·s^−1^)	2.872.35 ± 1,556.86	3.170.00 ± 1,464.65	3.221.38 ± 1,815.94	3.258.55 ± 1,994.01	3,116.87 ± 1,864.48	3,063.28 ± 1,723.86

Statistical analysis was performed using a two-way repeated measures analysis of variance, followed by Bonferroni *post hoc* pairwise comparisons. *Significant difference (*p* < 0.05) compared with baseline; **highly significant difference (*p* < 0.01) compared with baseline. Abbreviations: JH, jump height; PF, peak force; PP, peak power; ARFD, average rate of force development.

**FIGURE 5:**
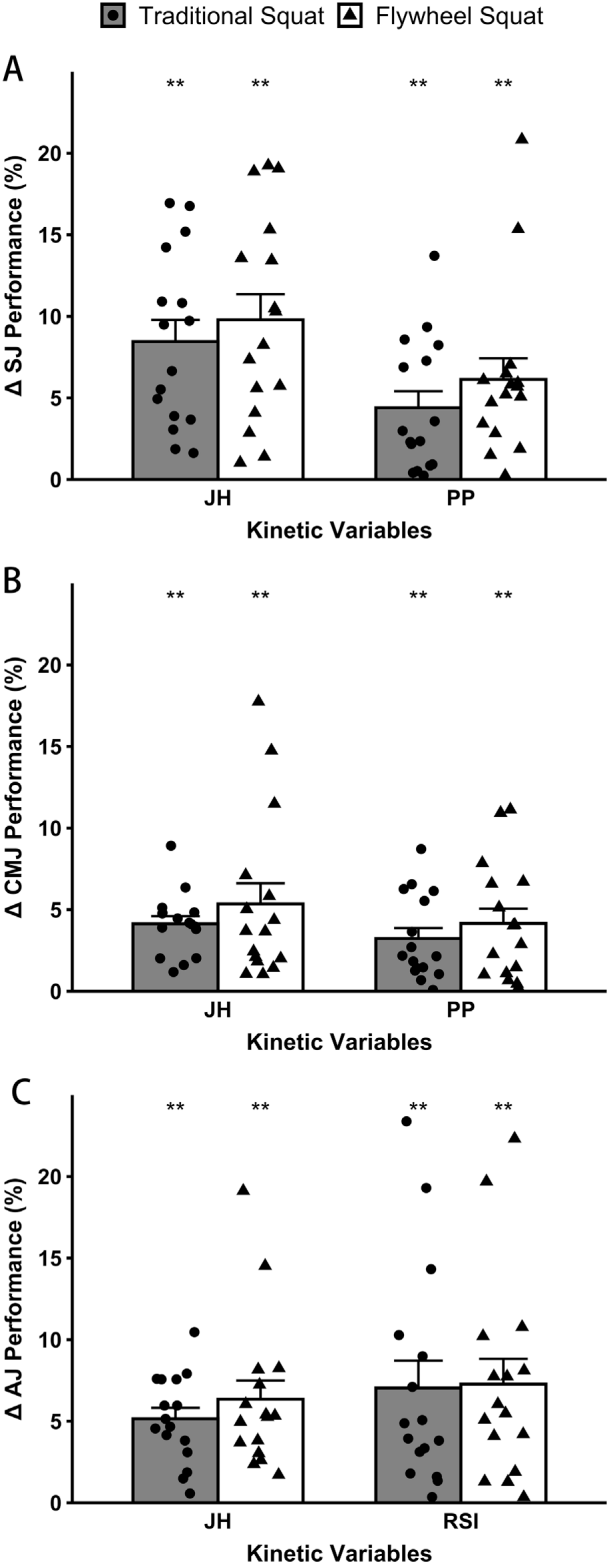
Percentage differences in the performance of squat jump **(A)**, countermovement jump **(B)**, and approach jump **(C)** for jump height (JH), peak power (PP), and reactive strength index (RSI) after implementing the individualized recovery time into both protocols. The error bars represent SE. **Highly significant difference (*p* < 0.01) compared with baseline within each protocol.

In the SJ, CMJ, and AJ tests, significant differences in JH, PP, and RSI were identified before and after implementing IRT into both protocols (Table 3), with all 16 participants exhibiting varying degrees of improvement above their baselines (Figures 5A–C). However, no significant differences were found between protocols (*p* ≥ 0.183). After the flywheel protocol, significant improvements were observed on JH in SJ, CMJ, and AJ (+9.79% [6.25%], +5.35% [5.07%], and +6.35% [4.61]; *p* < 0.001; 1.16 ≤ *d* ≤ 1.57), on PP in SJ and CMJ (+6.13% [5.16%] and +4.16% [3.60%]; *p* < 0.001; 1.17 ≤ *d* ≤ 1.23), and on RSI in AJ (+7.27% [6.22%]; *p* < 0.001; *d* = 1.19). Following the traditional protocol, significant increases were observed on JH in SJ, CMJ, and AJ (+8.46% [5.30%], +4.13% [1.91%], and +5.15% [2.68%]; *p* < 0.001; 1.67 ≤ *d* ≤ 2.26), on PP in SJ and CMJ (+4.40% [4.06%] and +3.23% [2.59%]; *p* ≤ 0.001; 1.10 ≤ *d* ≤ 1.27), and on RSI in AJ (+7.04% [6.71%]; *p* = 0.001; *d* = 1.07). No significant differences were found in PF and ARFD for both protocols when compared with baseline (*p* ≥ 0.08; 0.07 ≤ *d* ≤ 0.47).

**TABLE 3:** Effects of post-activation performance enhancement on jumping tests after implementing individualized recovery time into flywheel and traditional protocols (mean ± SD).

Jumping test	Variable	Flywheel protocol		Traditional protocol	
		Baseline	IRT	Baseline	IRT
Squat jump	JH (cm)	33.69 ± 4.77	36.86 ± 4.68 **	34.44 ± 4.91	37.25 ± 4.95 **
	PF (N)	2,209.42 ± 230.86	2,241.19 ± 208.85	2,177.36 ± 179.55	2,250.03 ± 217.94
	PP (W)	4,777.37 ± 572.42	5,069.94 ± 635.5 **	4,836.21 ± 544.17	5,045.29 ± 540.91 **
	ARFD (N·s^−1^)	6,572.9 ± 2,988.28	7,296.98 ± 3,656.88	6,048.63 ± 2,255.98	6,638.99 ± 1,971.89
Countermovement jump	JH (cm)	42.16 ± 4.27	44.33 ± 4.03 **	42.13 ± 3.84	43.85 ± 3.93 **
	PF (N)	2,040.37 ± 176.82	2,074.86 ± 168.28	2,055.09 ± 194.26	2,060.79 ± 182.83
	PP (W)	4,978.54 ± 601.74	5,185.69 ± 635.84 **	4,994.06 ± 620.26	5,154.08 ± 624.22 **
	ARFD (N·s^−1^)	5.334.5 ± 2,371.86	5,978.7 ± 2,816.0	5,306.8 ± 2510.72	5,696.51 ± 2278.9
Approach jump	JH (cm)	55.4 ± 4.53	58.88 ± 5.04 **	55.68 ± 5.20	58.51 ± 5.16 **
	PF (N)	2,650.31 ± 251.55	2,712.18 ± 276.51	2,646.82 ± 269.28	2,688.14 ± 254.17
	ARFD (N·s^−1^)	10,533.96 ± 3245.78	10,705.6 ± 3307.09	9,489.51 ± 4057.48	10,680.87 ± 3236.24
	RSI (m·s^−1^)	1.41 ± 0.21	1.51 ± 0.23 **	1.39 ± 0.21	1.49 ± 0.22 **

Statistical analysis was performed using a paired-sample *t*-test; **highly significant difference (*p* < 0.01) compared with baseline. Abbreviations: IRT, individualized recovery time; JH, jump height; PF, peak force; PP, peak power; ARFD, average rate of force development; RSI, reactive strength index.

## 4 Discussion

We investigated the effects of PAPE elicited by flywheel and traditional squats through WJS tests at baseline and at intervals of 2, 4, 6, 8, and 10 min. We also validated the effectiveness of implementing IRT into both protocols through SJ, CMJ, and AJ tests. Our findings indicated that both flywheel and traditional squats with high intensity can produce jumping potentiation, and the time courses were in a similar trend. For JH, PP, and RSI in WJS, SJ, CMJ, and AJ, the enhancement extent of the flywheel protocol was greater than that of the traditional protocol. After incorporating IRT, both protocols resulted in optimizing PAPE, with all participants making improvements of varying degrees.

### 4.1 Matching intensity by mean concentric linear velocity

Given the inherent reliance of flywheel training on concentric output, matching intensity with traditional training based on load alone could become problematic. Our findings revealed a significant reduction in MCLV as the inertial load increased (all *p* < 0.05). Additionally, a linear regression equation between MCLV and inertial load was statistically significant (*p* < 0.01) and strong, *R* ^2^ = 0.68 (equation: MCLV = −1.22 × IL + 0.87). To the best of our knowledge, the current study is the first to use VBT in analyzing each participant’s load–velocity profile to match the intensity of two potentiation protocols. This is in agreement with previous research supporting MCLV as an intensity prescription in FT. Carroll et al. (2019) found that the peak force, net impulse, and vastus lateralis muscle activation increased with the progressive inertial load and regressive mean concentric velocity. Similarly, Martín-Rivera et al. (2022) found that the RPE response, following a similar trend of increase, was significantly correlated with changes in inertial load and MCLV. In addition, some research workers proposed using peak concentric velocity as an intensity marker (McErlain-Naylor and Beato, 2021). However, it has shown less reliability across different devices. In contrast, the relationship between load and velocity is more linear when using mean velocity, and the between-subject variability in velocity attained during 1RM attempts may be lower (Weakley et al., 2021). Furthermore, the selection of the number of loads can also impact the validity and reliability of force–velocity outcomes in the flywheel squat. The study by Spudić et al. (2020) highlighted that employing four loads can reduce bias to 5% relative to the standard force–velocity slope. Additionally, future research is needed to adjust participants’ relative intensity in a dynamic training state.

### 4.2 Post-activation performance enhancement of flywheel and traditional squats

We utilized flywheel and traditional squats (8 repetitions) at 80% 1RM as potentiation protocols. In sessions involving WJS, SJ, CMJ, and AJ, significant increases were observed in JH, PF, PP, and RSI (*p* < 0.05; 0.90 ≤ *d* ≤ 2.26). The results indicated that both flywheel and traditional squats could induce a PAPE on vertical jumps with a large effect size, and flywheel squat potentially offered more benefits in specific neuromechanical metrics (JH: 5.35%–9.79% vs. 4.13%–8.46%; PP: 4.16%–6.13% vs. 3.23%–4.77%; RSI: 7.27% vs. 7.04%). In keeping with previous studies, a worthwhile increase in the vertical jump can be achieved by following a biomechanically similar squat when using an intensity ≥80% 1RM (Robbins, 2005; Suchomel et al., 2016). Given the extensive knowledge of traditional exercise on PAPE, comparing FT to such exercises could further highlight flywheel’s effectiveness in inducing PAPE. However, the correlation between the effects of PAPE and changes in physiological indicators (e.g., muscle temperature, muscle water content, and muscle–tendon mechanical properties) have not been clearly defined (Blazevich and Babault, 2019; Krzysztofik et al., 2023a). It is believed that the benefits of FT stem from the unique response of eccentric resistance training, including a greater capacity and a lower metabolic cost per unit at the same intensities as concentric action. This energetic advantage may optimize the balance between PAPE and fatigue, making it easier for PAPE to be dominant. A further advantage of FT as conditioning activities is the consistent greater eccentric force, power, and derivative outputs produced, potentially leading to more transfer effects on rapid, mixed eccentric/concentric movements (Beato et al., 2020). Moreover, the point of force application in traditional squats is the trapezius muscle and that of a flywheel squat is the lower back muscle. This implies that traditional squats would activate upper body muscles more, which may lead to unfavorable outcomes of lower body enhancement. Therefore, there is a need to use more specific and localized exercises to produce PAPE (Kolinger et al., 2024).

Similar to the role of gravitational load in traditional protocols, the size of the inertial load can influence the magnitude of the PAPE effect induced by flywheel protocols. Fu et al. (2023) demonstrated this by using three different inertial loads as CAs. Their findings showed that larger inertial loads can significantly improve acute lower limb explosive performance, particularly in CMJ test. However, it should be noted that due to the properties of flywheel devices, eccentric mechanical stress does not necessarily correlate with inertial load or MCLV, and small inertial loads can also produce significant activation effects. In contrast, using gravitational exercises to induce PAPE typically requires near-maximal loads (Seitz and Haff, 2016). Timon et al. (2019) found that performing high-power flywheel squats with relatively light loads could produce significant acute enhancement on SJ performance, whereas traditional squats did not. Thus, the flywheel squat might be particularly effective in inducing PAPE at lighter loads, offering advantages for load-compromised individuals and other special populations. This also emphasizes the versatility of implementing eccentric training. According to the research of Burgos-Jara et al. (2023), eccentric training methods can be flexibly adjusted based on training objectives (e.g., energy attenuation for protection and energy storage for potentiation), differing in movement complexity, intensity, volume, technology, and time under tension. It would be worth to explore other flywheel protocol modalities to induce PAPE, including variations in intensity, volume, and movement type (e.g., horizontal plane).

We observed no significant interactional effects between the protocol and time variables, with recovery curves after both protocols displaying a similar inverted U-shaped pattern, peaking between 2 and 6 min. According to the theoretical model of the interaction between fatigue and potentiation (Tillin and Bishop, 2009; Sale, 2002), this may be attributed to the similar trends of the two protocols on the generation of PAPE and the dissipation of fatigue. Our findings were basically identical with the research by Dobbs et al. (2019), suggesting that potentiation for trained individuals is optimal with a dynamic movement at 80% 1RM, followed by a 3- to 7-min rest period; the slight difference in the optimal window of PAPE response could be attributed to the unique physiological adaptations of our participants, who are well-trained volleyball players accustomed to quickly recovering from intense exercises. Moreover, due to the specialization of the field position in team sport athletes, the differences in specialized training experience also could lead to variations in the PAPE response. Consequently, sports with specific metabolic requirements and athletes from diverse training backgrounds often exhibit varying optimal recovery periods for PAPE (Kilduff et al., 2007; Seitz et al., 2014).

### 4.3 Individualizing recovery time for optimizing post-activation performance enhancement

The WSJ results showed that the peak performance enhancement on JH and PP showed a large effect size and statistical significance (0.92 ≤ *d* ≤ 1.23; *p* < 0.05; 42.00 ≤ CV% ≤ 43.49) after implementing both traditional and flywheel protocols. We observed highly significant improvements in JH, PP, and RSI using IRT in the protocols, with every participant showing varying degrees of improvement from their baseline levels. The JH and PP enhancement in SJ, CMJ, and AJ sessions showed a larger effect size, higher statistical significance, and a lower coefficient of variation (1.10 ≤ *d* ≤ 2.26; *p* < 0.01; 15.92 ≤ CV% ≤ 22.75). Our findings are in agreement with those of the previous study that recommends customizing the intra-complex recovery time for competitive athletes, likely through the trial and error method, with rest intervals from 2 to 10–12 min (Gołaś et al., 2016). Many research workers have attempted to establish theoretical frameworks that offer guidelines for manipulating factors to optimize PAPE (Dobbs et al., 2019; Gouvêa et al., 2013; Suchomel et al., 2016), yet practical applications show considerable variations in individual responses. In our study, we identified each participant’s IRT based on the rest periods of the best trials during WJS sessions and subsequently applied this IRT to SJ, CMJ, and AJ sessions. These results suggest that implementing a uniform recovery time can significantly potentiate jumping performance, reduce inter-individual variability in responses, and enhance the activation effects more substantially.

Additionally, the observed variations in the effects of IRT highlight the necessity of incorporating more customized factors. We also observed differences among participants in the force–velocity curve, indicating a requirement for targeted attention within the force–velocity spectrum (e.g., maximum strength, speed-strength, and speed) to elicit further enhancements in jumping performance (Brady et al., 2017). According to the research conducted by Krzysztofik et al. (2023b), applying plyometric CAs instead of high-loaded CAs contributes to achieving more PAPE-positive responders in athletes with low lower-muscle strength. Therefore, due to the high intra-individual and inter-individual variability of the recovery processes, optimizing performance effectively necessitates a systematic and comprehensive approach to monitor PAPE–fatigue continuum and customize training and recovery strategies.

### 4.4 Limitations and future perspectives

This study is not without limitations. First, we enrolled well-trained male volleyball players with a minimum of 4 years of resistance training experience. Therefore, caution should be exercised when generalizing these results to other populations and conditions. Additionally, our participants attended the laboratory over a 5- to 6-week period, during which neuromuscular adaptations could occur, potentially introducing biases or errors in the results. In addition, the ideal intensity based on velocity should be dynamically adjusted to account for fluctuations in the participants’ conditions. Lastly, future studies should include a more detailed examination of the effects of individual variability and their impact on the observed PAPE effect, especially considering that the characteristics of force–velocity profiles may imply potential improvements.

## 5 Conclusion

This study was the first to apply the concept of VBT to compare flywheel and traditional protocols at matching intensity. We discovered that both flywheel and traditional squats performed at high intensity could enhance acute jumping performance and displayed comparable recovery patterns. However, the flywheel squats demonstrated greater enhancement in specific neuromechanical parameters (such as JH, PP, and RSI), making them a more practical option for inducing PAPE. Meanwhile, applying IRT into potentiation protocols could further optimize the effects of PAPE, which may contribute to the development of customized training programs. Practitioners may consider dynamically monitoring athletes’ responses with VBT devices and customizing PAPE recovery time individually to optimize jumping potentiation.

## Data Availability

The raw data supporting the conclusions of this article will be made available by the authors, without undue reservation.
